# Metal-Doped NASICON/Polymer Composite Solid Electrolyte for Lithium Titania Anode in Lithium-Ion Batteries

**DOI:** 10.3390/polym16091251

**Published:** 2024-04-30

**Authors:** Chien-Te Hsieh, Tzu-Shaing Cho, Jeng-Kuei Chang, Jagabandhu Patra

**Affiliations:** 1 Department of Chemical Engineering and Materials Science, Yuan Ze University, Taoyuan 32003, Taiwan; 2 Department of Mechanical, Aerospace, and Biomedical Engineering, University of Tennessee, Knoxville, TN 37996, USA; 3 Hierarchical Green-Energy Materials (Hi-GEM) Research Center, National Cheng Kung University, 1 University Road, Tainan 70101, Taiwan; 4 Department of Materials Science and Engineering, National Yang Ming Chiao Tung University, Hsinchu 30010, Taiwan; 5 Department of Chemical Engineering, Chung Yuan Christian University, Taoyuan 32023, Taiwan

**Keywords:** cation doping, solid-state batteries, electrolyte conductivity, rate capability, cycle life

## Abstract

This study reports five types of metal-doped (Co, Cu, Sn, V, and Zr) NASICON-type Li_1.3_Al_0.3_Ti_1.7_(PO_4_)_3_ (LATP)/polymer composite solid electrolytes (CSEs) enabling Li_4_Ti_5_O_12_ (LTO) anodes to have high rate capability and excellent cycling performance. The high Li^+^-conductivity LATP samples are successfully synthesized through a modified sol–gel method followed by thermal calcination. We find that the cation dopants clearly influence the substitution of Al for Ti, with the type of dopant serving as a crucial factor in determining the ionic conductivity and interfacial resistance of the solid electrolyte. The CSE containing poly(vinylidene fluoride-co-hexafluoropropylene) (PVDF-HFP), lithium bis(trifluoromethanesulfonyl)imide (LiTFSI), and Sn-LATP shows an ionic conductivity of 1.88 × 10^−4^ S cm^−1^ at ambient temperature. The optimum conductivity can be attributed to alterations in the lattice parameters and Li^+^ transport pathways owing to Sn doping. The solid-state cell equipped with the LTO-supported CSE containing Sn-LATP fillers demonstrates both excellent high rate capability at 5 C (with a capacity retention of 86% compared to the value measured at 0.2 C) and superior cycling stability, maintaining high Coulombic efficiency (>99.0%) over 510 cycles. These findings indicate that the proposed CSE is highly promising for use in solid-state lithium batteries with desirable charge–discharge properties and high durability.

## 1. Introduction

The importance of lithium-ion batteries (LIBs) has been acknowledged over several decades; nevertheless, continuous endeavors persist to enhance both the performance and safety of LIBs. Meeting the demands of LIB applications, which span from miniaturized electronics and home appliances to light and heavy electric vehicles [1,2,3,4], necessitates achieving a high energy density and a high power density. The energy density of LIBs is directly dominated by the redox potential difference between the positive (cathode) and negative (anode) electrodes [5], as well as their capacities. Traditionally, graphite has been the favored material for LIB anodes. The primary advantage of graphite lies in its low intercalation potential (~0.1 V vs. Li^+^/Li) and low cost [6]. Lithium titanate (Li_4_Ti_5_O_12_, LTO) has emerged as a promising substitute for traditional graphite anode materials for LIBs. The LTO anode operates at a higher potential (~1.55 V vs. Li^+^/Li), offering enhanced safety features due to the prevention of Li dendrite formation [7]. The LTO anode has demonstrated remarkable cycling stability owing to its minimal volume change during Li^+^ intercalation/deintercalation. Additionally, LTO has exhibited superior stability at high working temperatures and a greater rate capability compared to that of graphite anodes [8], which is favorable for the high-power performance of LIBs.

The utilization of conventional LIBs that incorporate organic liquid electrolytes (i.e., volatile and flammable carbonate electrolytes) poses the risk of thermal instability, leading to potential explosion and catastrophic fire [9,10]. Consequently, solid-state lithium batteries (SSLBs) are considered promising next-generation lithium batteries. Solid-state electrolytes (SSEs), chosen over their liquid counterparts, mitigate safety concerns, and, importantly, these electrolytes can be coupled with various anode and cathode materials with desirable potentials and capacities for LIBs [11,12,13,14]. SSEs can be broadly categorized into two types: solid polymer electrolytes (SPEs) and inorganic solid (i.e., ceramic) electrolytes (ISEs) [15,16]. The poor ionic conductivity, inferior mechanical properties, and inadequate oxidation stability limit the use of SPEs [15], while ISEs are usually fragile and challenging to fabricate, making them unsuitable for practical applications [16]. Composite solid electrolytes (CSEs), composed of a polymer phase, Li salt, and Li^+^-conducting inorganic fillers, have become a promising alternative [17,18]. In CSEs, the polymer phase has good flexibility, plasticity, and wettability toward electrodes [19,20], while the ceramic filler imparts high Li^+^ conductivity and boosts the mechanical strength to prevent electrode short-circuiting [21,22]. A CSE, thus, blends the merits of SPEs and ISEs, endowing SSLBs with desirable charge–discharge properties. In fact, inorganic filler materials play a crucial role in CSEs. Among various inorganic Li^+^-conducting fillers, NASICON-type Li_1.3_Al_0.3_Ti_1.7_(PO_4_)_3_ (LATP) [23] and garnet-type Li_7_La_3_Zr_2_O_12_ (LLZO) have received a considerable amount of attention due to their adequate Li^+^ conductivity and good chemical stability [24]. LLZO is chemically stable against Li metal [25]. LATP possesses a significant advantage in terms of material synthesis and practical commercialization, considering the relatively low cost of the raw materials and fabrication process. The high stability of LATP against H_2_O/O_2_ allows the material preparation and handling to proceed in the ambient air [23,26]. 

The exploration of LTO-supported SSEs for SSLBs remains limited, with only a few studies delving into this topic. Investigating LTO-supported SSEs has the potential to yield significant advancement in SSLB performance. The integration of LTO as a supporting material for SSE can enhance the overall stability, rate capability, and cycling performance of the battery. Moreover, the adoption of LTO-supported SSEs may play a pivotal role in addressing the key challenges associated with SSLBs, including high interfacial resistance and high SSE thickness [27,28,29]. Exploring the properties offered by LTO-supported SSE can pave the way for the development of high-performance SSLBs characterized by improved safety and stability. 

One approach to enhance the ionic conductivity of LATP-based SSEs is through element doping [30,31,32,33], which involves partially replacing Ti^4+^ ions with metal ions. The LATP framework can be modified by introducing foreign cations with different valence states and ion radii, which causes lattice distortion and enhances ionic conductivity. This present work aims to develop an efficient sol–gel synthesis method for producing metal-doped Li_1.3_Al_0.3_(Ti_1.7−*x*_M*_x_*)(PO_4_)_3_, where Ti is partially replaced by other elements (M) such as Co, Cu, Sn, V, and Zr with *x* = 0.3. These elements are the most common dopants for battery electrodes and solid electrolyte materials, and the systematic comparison of the doping effects in the LATP lattice has never been conducted. In the sol–gel process, anhydrous ethanol is used to prevent the hydrolysis of the Ti precursor (i.e., titanium (IV) butoxide) and shorten the reaction time. In this study, we develop CSEs incorporating various metal-doped LATP fillers within polymeric matrixes for application in SSLBs. The CSE consists of metal-doped LATP powder, poly(vinylidene fluoride-co-hexafluoropropylene) (PVDF-HFP), and lithium bis(trifluoromethanesulfonyl)imide (LiTFSI). Although some studies have utilized polyethylene oxide (PEO), PEO-based systems tend to crystallize at ambient temperature, which can hinder ionic transport. PVDF-HFP is preferred over PEO due to its superior electrochemical stability and processability, as well as its chemical and mechanical stability across a broader temperature range [34,35]. Furthermore, HFP serves as an amorphous phase promoter, offering ion channels that increase the ionic conductivity of the electrolyte system [35]. We conduct a detailed investigation into the impact of these CSEs on the specific capacity, rate capability, and cycling stability of Li||CSE||LTO cells. The excellent compatibility of the CSEs with LTO anodes secures a robust battery structure with low internal resistance and superior capacity retention during extended cycling. 

## 2. Experimental Section

### 2.1. Sol–Gel Synthesis of Metal-Doped LATP Powders

The sol–gel synthesis of metal-doped Li_1.3_Al_0.3_(Ti_1.7−*x*_M*_x_*)(PO_4_)_3_ was carried out by replacing Ti with other M elements, where *x* = 0.3 and M = Co, Cu, Sn, V, and Zr. The synthesis procedures for metal-doped LATP powders are outlined as follows. All reagents employed in this study are of an analytical grade. A Ti(C_6_H_9_O)_4_ (Sigma-Aldrich (St. Louis, MO, USA)); purity: 97%) precursor was dissolved in anhydrous ethanol. Stoichiometric amounts of LiNO_3_ (J.T. Baker (Phillipsburg, NJ, USA)); purity: 98%) and Al(NO_3_)_3_·9H_2_O (Sigma-Aldrich; purity: 99.9%) were dissolved in a 0.2 M citric acid (Showa (Gyoda, Japan); purity: 99.5%) solution and stirred at 90 °C for 1 h to form a stable and clear solution. Simultaneously, various metal ions were separately introduced into the solution with a stoichiometric ratio of Ti:M (1.4:0.3), where M = Co, Cu, Sn, V, and Zr. The Co(NO_3_)_2_ (Alfa Aesar (Haverhill, MA, USA)); purity: 98%), CuSO_4_·5H_2_O (Alfa Aesar; purity: 98%), SnCl_2_ (Alfa Aesar; purity: 99%), VCl_3_ (Sigma-Aldrich; purity: 97%), and ZrCl_4_ (Sigma-Aldrich; purity: 99.9%) precursors were used. Subsequently, step-wise citric acid addition was performed to maintain a molar ratio of citric acid to total metal ions at 4:1. After adding a saturated NH_4_H_2_PO_4_ (Alfa Aesar; purity: 99.995%) solution in a stoichiometric amount, the pH value of the mixed solution was adjusted to 7 using NH_3_·H_2_O. After maintaining at 90 °C for 5 h, the solution became a homogeneous emulsion, which was then moved to an oven at 120 °C until a dry gel was produced. The obtained dry gel was heated to 180 °C for 4 h and then 250 °C for 5 h to proceed with pyrolysis. The metal-doped LATP samples were eventually calcined at 850 °C under air for 2 h, while the temperature ramping rate was 5 °C min^−1^. Through ball-milling with a planetary mill (Fritsch Pulverisette 6 (Idar-Oberstein, Germany)) at 700 rpm for 90 min with 1 mm diameter ZrO_2_ balls (the ball to LATP weight ratio is 1:15), highly refined metal-doped LATP particles were obtained. The resulting metal-doped LATP samples were designated as Co-LATP, Cu-LATP, Sn-LATP, V-LATP, and Zr-LATP, according to the type of dopants. Pristine LATP powder without any dopants was designated as 0-LATP for comparison. 

### 2.2. Fabrication of CSEs on LTO Anode Sheets

Anode slurry was prepared by mixing 88 wt% LTO powder, 6 wt% PVDF binder, 4 wt% Super-P, and 2 wt% KS-6 (conducting agent) in *N*-methyl-*2*-pyrrolidone (NMP) solution. This slurry was casted onto Cu foil with a doctor blade and vacuum-dried at 90 °C for 12 h. The coating layer was ~100 μm in thickness. The LTO anode sheets were roll-pressed and then cut into the desired dimensions for battery assembly. To fabricate the LTO-supported CSEs, LiTFSI (Sigma-Aldrich; purity: 99.95%) and PVDF-HFP (Sigma; molecular weight: 400,000) were dissolved in NMP and stirred well to form a homogeneous solution. The metal-doped LATP powder was gradually added to the solution and the resulting slurry was uniformly dispersed through a planetary milling process with a rotation speed of 700 rpm for 1 h. The obtained CSE slurry was then coated onto the LTO anode sheet using a doctor blade. The sheets were subjected to drying at 140 °C under vacuum overnight to form LTO-supported CSE samples. The thickness of the CSE layers was ~40 μm.

### 2.3. Material and Electrochemical Characterizations

The morphology of the metal-doped LATP samples was analyzed using field-emission scanning electron microscopy (FE-SEM; Zeiss ULTRA 55 (White Plains, NY, USA)) and transmission electron microscopy (TEM; FEI Talos F200s (Hillsboro, OR, USA)). The sample crystallinity was examined using X-ray diffraction (XRD; Bruker D2 Phaser (Billerica, MA, USA)). For electrochemical property measurements of LTO-supported CSE samples, coin cells of CR2032 type were utilized with Li metal foil as the counter electrodes. The coin cells were assembled in an argon-filled glove box (Innovation Technology Co., Ltd. (Hong Kong, China)), where both the moisture and oxygen content levels were maintained at ~0.1 ppm. The charge–discharge capacities, rate capability, and cycling stability were evaluated at ambient temperature. The durability of the cells was evaluated through repeated cycling at 0.2 C charging and 0.5 C discharging; once the capacity retention degraded to <80%, the testing was terminated. The internal resistance of the coin cells assembled with various LTO-supported CSEs was evaluated using electrochemical impedance spectroscopy (EIS; CH Instruments 608C (Bee Cave, TX, USA)) within a frequency range of 100 kHz to 10 mHz. 

## 3. Results and Discussion

**Figure 1:**
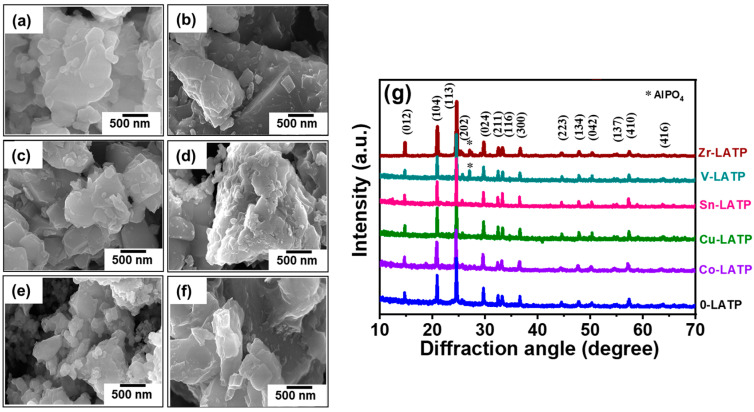
SEM images of (**a**) 0-LATP, (**b**) Co-LATP, (**c**) Cu-LATP, (**d**) Sn-LATP, (**e**) V-LATP, and (**f**) Zr-LATP samples. (**g**) XRD pattern of various samples synthesized.

Figure 1a–f show the FE-SEM images of pristine and metal-doped LATP powders. A slight degree of agglomeration is apparent in the pristine and metal-doped LATP powders, indicating a tendency of the particles to cluster together to some extent without substantial interconnection. The images reveal that the particle sizes of the samples range from 1.2 to 2.5 μm. This size distribution reveals the decent homogeneity of the particles. Figure 1g exhibits the XRD patterns of various LATP powders, which were prepared using the citric-acid-assisted sol–gel synthesis route followed by thermal calcination. The XRD pattern of the pristine LATP sample aligns with the standard NASICON-type structure (i.e., rhombohedral lattice; Card No.: ICDD 00-035-0754) [36]. The major characteristic peaks at the diffraction angles of 20.8°, 24.5°, 29.7°, and 33.3° correspond to the crystalline planes of (104), (113), (024), and (116), respectively [37,38]. Notably, there is an absence of any impurity phase in this pristine LATP sample. The metal-doped LATP samples demonstrate the same rhombohedral structure, although a minor impurity phase of AlPO_4_ [30,33] is observed for the V-LATP and Zr-LATP samples. This observation implies that the V and Zr dopants could easily cause lattice distortion, leading to the extraction of aluminum atoms from the lattice to form AlPO_4_.

**Figure 2:**
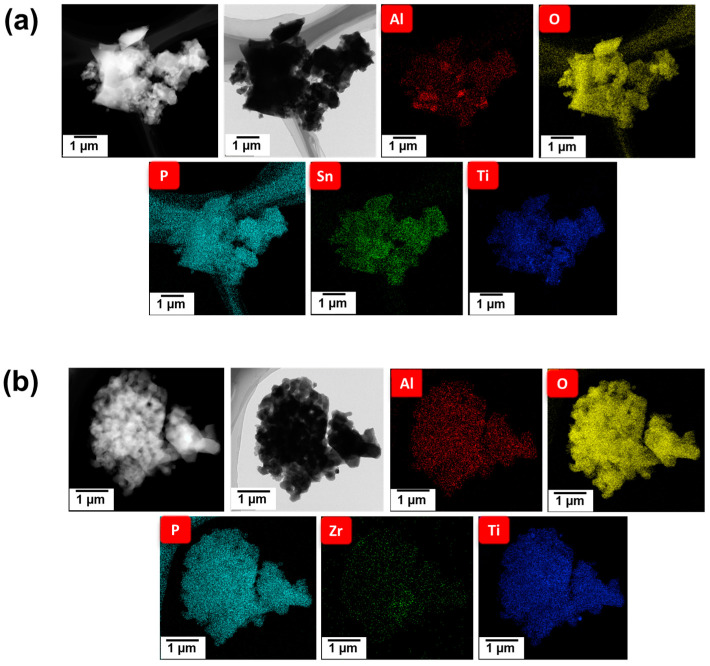
TEM micrographs of (**a**) Sn-LATP and (**b**) Zr-LATP samples and their corresponding elemental mapping data.

Figure 2a,b illustrate the TEM micrographs of the Sn-LATP and Zr-LATP samples together with their elemental mapping data. Notably, these particles exhibit irregular morphology with a diameter of up to a few microns. Elements such as Al, Ti, P, and O are discernible across the metal-doped LATP samples. Of note, both the Sn and Zr dopants are uniformly dispersed within the particles. The energy-dispersive spectroscopy data also confirmed that the M concentrations are 11.1 at%, 16.4 at%, 17.0 at%, 10.8 at%, and 11.5 at% for Co-LATP, Cu-LATP, Sn-LATP, V-LATP, and Zr-LATP samples, respectively.

**Figure 3:**
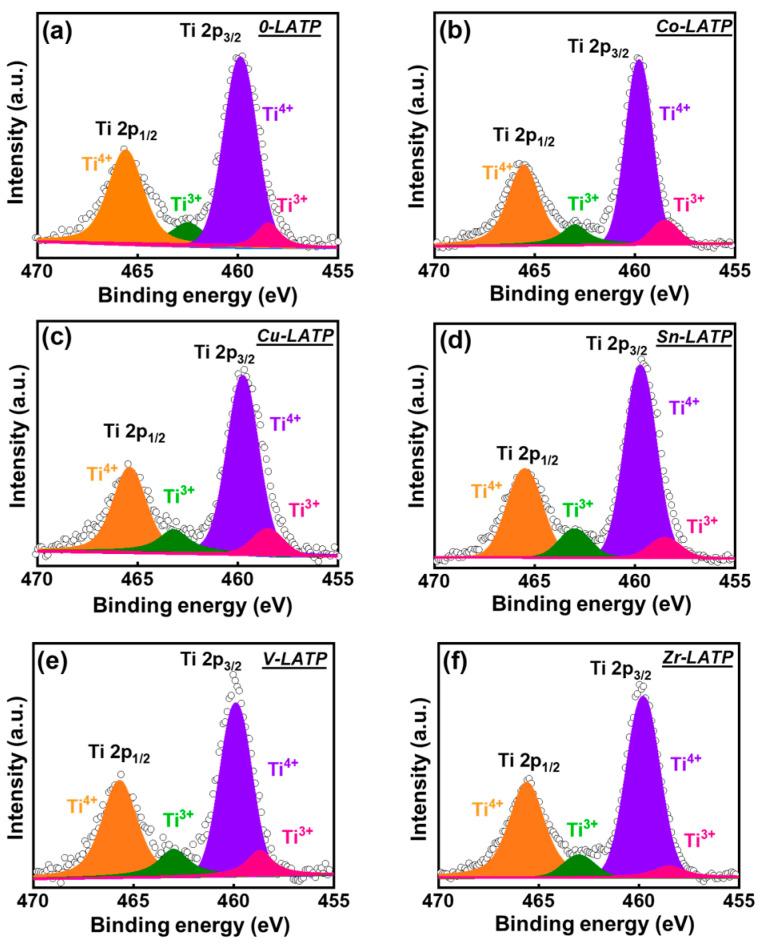
High-resolution XPS Ti 2p spectra of (**a**) 0-LATP, (**b**) Co-LATP, (**c**) Cu-LATP, (**d**) Sn-LATP, (**e**) V-LATP, and (**f**) Zr-LATP samples.

**Figure 4:**
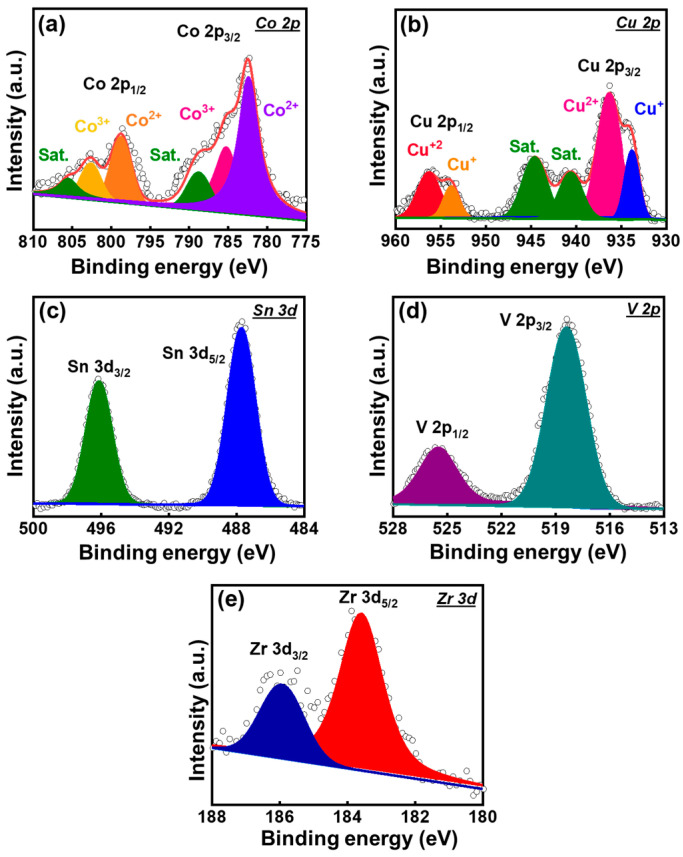
High-resolution XPS (**a**) Co 2p, (**b**) Cu 2p, (**c**) Sn 3d, (**d**) V 2p, and (**e**) Zr 3d spectra for Co-LATP, Cu-LATP, Sn-LATP, V-LATP, and Zr-LATP samples, respectively.

The oxidation states of Ti in the pristine and metal-doped LATP samples were investigated using X-ray photoelectron spectroscopy (XPS). The obtained data are shown in Figure 3. The two prominent peaks at approximately 459.6 and 465.5 eV correspond to Ti 2p_3/2_ and Ti 2p_1/2_, respectively [39,40]. As shown, for all of the LATP samples, Ti^4+^ is the major component, which is consistent with the literature [39,40,41]. However, two shoulder peaks at lower binding energies indicate the presence of Ti^3+^ [32,42]. The Ti^4+^/Ti^3+^ ratios for various samples do not show significant difference. The high-resolution Co 2p, Cu 2p, Sn 3d, V 2d, and Zr 3d spectra for the Co-, Cu-, Sn-, V-, and Zr-LATP samples are shown in Figure 4. The Co 2p spectrum (Figure 4a) shows Co 2p_3/2_ and Co 2p_1/2_ signals [43]. Two satellite peaks are at 788.7 and 805.6 eV. The data show the coexistence of Co^2+^ and Co^3+^. The Cu 2p spectrum (Figure 4b) shows two spin-orbital signals of Cu 2p_3/2_ and Cu 2p_1/2_ [44]. The co-existence of Cu^+^ and Cu^2+^ are confirmed. The two satellite peaks of Cu 2p_3/2_ are centered at 940.0 and 944.6 eV, respectively. In the Sn 3d spectrum (Figure 4c), the peaks at 487.7 eV (Sn 3d_5/2_) and 496.1 eV (Sn 3d_3/2_) belong to Sn^4+^ [45]. Figure 4d shows the characteristic peaks at 518.3 and 525.5 eV, which are ascribed to V 2p_3/2_ and V 2p_1/2_ of V^5+^, respectively [46]. The Zr 3d spectrum in Figure 4e shows the characteristic signals of Zr 3d_5/2_ and Zr 3d_3/2_ at 185.8 and 183.6 eV, respectively. Both peaks belong to Zr^4+^ [47]. The data confirm that the cations are indeed doped in the LATP lattice.

**Figure 5:**
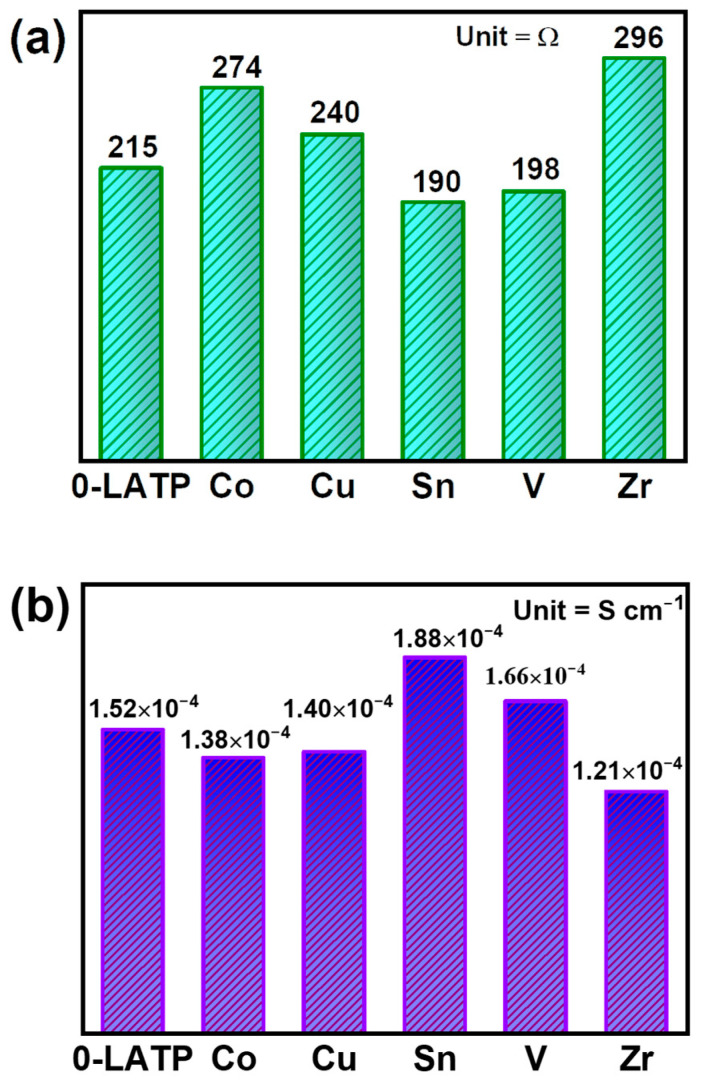
(**a**) ESR values of various Li||CSE||LTO cells. (**b**) Li^+^ conductivity values of various types of CSE.

The EIS measurements were carried out with Li||CSE||LTO cells and conducted at ambient temperature in a frequency range of 100 kHz to 10 mHz. The representative data and the equivalent circuit used to fit the data are shown in Figure S1 (supporting information). The equivalent series resistance (ESR = resistance of bulk electrolyte + electrolyte/electrode interfacial resistance *+* charge-transfer resistance) was assessed to determine the overall internal resistance of the cells [48]. As shown in Figure 5a, the ESR values of the cells exhibit a strong dependence on the type of dopant. The order of ESR values is as follows: Zr -LATP (296 Ω) > Co-LATP (274 Ω) > Cu-LATP (240 Ω) > 0-LATP (215 Ω) > V-LATP (198 Ω) > Sn-LATP (195.5 Ω). These results highlight that the ESR value, representing the overall cell resistance, is significantly reduced for the cells containing the Sn-LATP and V-LATP CSEs.

The ionic conductivity (*σ*) of various CSEs can be quantified to clarify the effects of the design of LATP powders by utilizing the high-frequency impedance (*R*) obtained from the EIS analysis. The ionic conductivity is determined using the formula *σ* = *l*/(*R* × *A*), where *l* is the thickness of the CSE and *A* is the projected area [49,50]. This calculation allows for the evaluation of the ionic conductivity of various CSEs. As depicted in Figure 5b, the *σ* values of the CSE samples follow the order of Sn-LATP (1.88 × 10^−4^ S cm^−1^) > V-LATP (1.66 × 10^−4^ S cm^−1^) > 0-LATP (1.52 × 10^−4^ S cm^−1^) > Cu-LATP (1.40 × 10^−4^ S cm^−1^) > Co-LATP (1.38 × 10^−4^ S cm^−1^) > Zr-LATP (1.21 × 10^−4^ S cm^−1^). These results highlight the clear improvement in ionic conductivity achieved through the chemical composition design of the ceramic fillers. As compared to the *σ* value of the LATP-0 sample, the Sn and V dopants in the LATP lattice show positive effects in increasing the ionic conductivity of the CSEs. Numerous factors influence the ionic conductivity of the doped LATP, including the dopant size, electronegativity, lattice volume, Li^+^ concentration, lattice vacancies, and the interactions with the polymer phase [42,51,52]. Consequently, it is challenging to precisely determine which factor dominates the conductivity of Sn-LATP at the current stage. Although we have experimentally examined the order of conductivity, the exact causes and underlying mechanisms for the superior conductivity observed still require further investigation.

**Figure 6:**
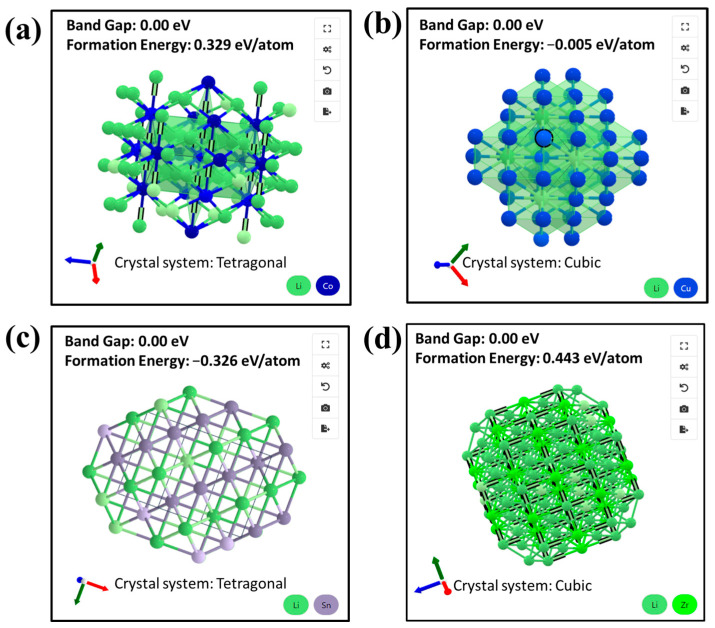
Schemes of bonding structures of (**a**) Li-Co, (**b**) Li-Cu, (**c**) Li-Sn, and (**d**) Li-Zr systems and their corresponding formation energies.

The experimental results presented above indicate that the metal dopants for LATP play a significant role in enhancing the ionic conductivity of the solid electrolyte. Nevertheless, a more comprehensive investigation is needed to further understand how dopants such as Sn and V contribute to the improvement in Li^+^ conduction in the LATP lattice. Theoretically, three primary Li^+^ migration mechanisms in the crystal may be involved: (i) the ion migrating directly from one lattice site to a neighboring vacant site, (ii) the ion migrating from one site to a neighboring metastable vacant site through an interstitial site, and (iii) the ion migrating into a neighboring metastable vacant site followed by the occupation of its original site by an ion from the neighboring metastable site [53]. Based on these migration mechanisms, the Sn and V dopants are likely to create vacant sites (or grain boundaries), promoting ionic migration in the lattice and thereby enhancing Li^+^ conductivity. This leads to a unique ion transport mechanism where Li^+^ ions hop from one coordinating site to another, facilitated by the movement of defect sites, similar to the studies reported previously [12,54,55]. Figure 6 illustrates the bonding structures of Li-Co, Li-Cu, Li-Sn, and Li-Zr with the corresponding formation energies, as programmed by The Materials Project [56,57,58]. Particularly noteworthy is the most negative formation energies of the Li-Sn bonding structure (i.e., −0.326 eV/atom), indicating the most favorable formation relative to their bonds. This suggests that the Li^+^ conducting pathways are relatively easy to form for Sn-LATP, leading to its superior ionic conductivity.

**Figure 7:**
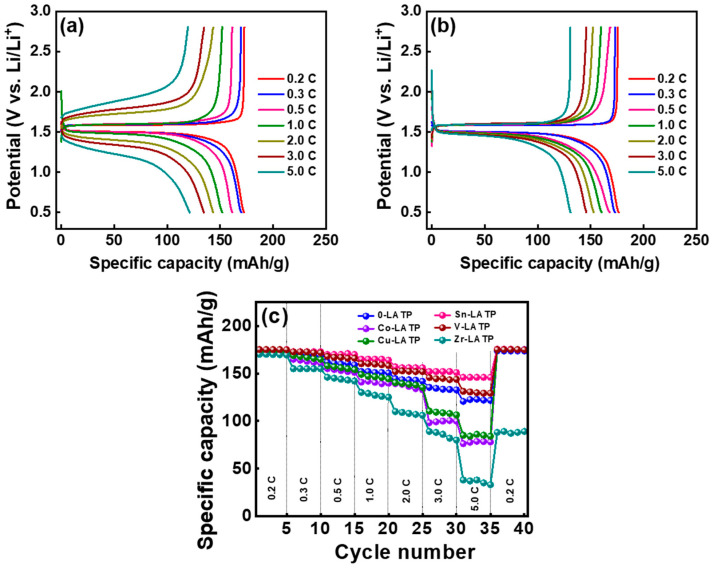
Charge–discharge curves of LTO half cells equipped with (**a**) 0-LATP and (**b**) Sn-LATP CSEs. (**c**) Comparative rate performance of various cells.

The charge–discharge profiles of the LTO half cells equipped with the CSEs containing 0-LATP and Sn-LATP are shown in Figure 7a,b, respectively. The charging and discharging tests were carried out within a voltage range of 0.5 to 2.8 V (vs. Li/Li^+^) at different rates ranging from 0.2 to 5 C. At 0.2 C, the cells exhibit distinct voltage plateaus, signifying a two-phase reversible reaction at approximately 1.5 V vs. Li/Li^+^. The intercalation/de-intercalation process can be expressed as [7] Li_3_[LiTi_5_^4+^]O_12_ + 3 e^−^ + 3 Li^+^ ↔ Li_6_[LiTi_3_^3+^Ti_2_^4+^]O_12_. During the electrode lithiation process, the Li ions insert into the octahedral (16c) sites of Li_4_Ti_5_O_12_ lattices, forming a rock-salt structure [8]. Theoretically, a typical LTO electrode offers a reversible capacity of 175 mAh g^−1^, based on the Li^+^ insertion/de-insertion process mentioned above. Figure 7c compares the rate capability of various cells across the range of 0.2–5 C. As evident from the plot, all of the LTO electrodes exhibit high specific capacities of ~170 mAh g^−1^ at 0.2 C, very close to the theoretical value. At a high rate of 5 C, the cells with CSEs containing 0-LATP, Co-LATP, Cu-LATP, Sn-LATP, V-LATP, and Zr-LATP fillers showed reversible capacities of 120, 76, 85, 146, 131, and 38 mAh g^−1^, respectively, corresponding to 70%, 44%, 49%, 86%, 77%, and 23% capacity retention compared to the values measured at 0.2 C. When the current rate decreased back to 0.2 C, the capacities were restored, as shown in Figure 7c. The cells equipped with the CSEs containing Sn-LATP and V-LATP demonstrated superior rate capability owing to the relatively low ESR and high Li^+^ conductivity of the electrolyte layers. This promising outcome (i.e., high electrode performance at high rate) underscores that the LTO-supported CSEs have paved the way for realizing a high-power SSLB.

**Figure 8:**
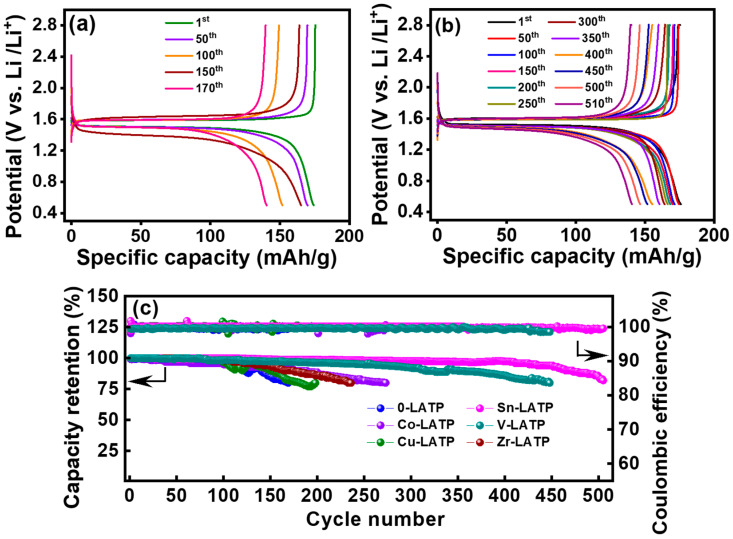
Charge–discharge curves of LTO half cells equipped with (**a**) 0-LATP and (**b**) Sn-LATP CSEs upon cycling. (**c**) Cycling stability data of various cells until capacity retention of 80%.

Figure 8a,b compare the charge–discharge curves of the LTO cells equipped with the CSEs containing 0-LATP and Sn-LATP fillers, respectively. The objective of this experiment was to assess the electrochemical stability of the cells through galvanostatic cycling at ambient temperature. An observable plateau at ~1.5 V and stable discharge performance upon cycling for the Sn-LATP cell reflect the robustness of the CSE layer. In addition, the high reversibility of Li^+^ intercalation and de-intercalation into/from the spinel LTO structure was confirmed. Figure 8c shows the capacity retention and Coulombic efficiency as a function of cycle number for various LTO-supported CSE cells. These metrics serve as indicators to assess the cycling stability of the cells. The cycling tests continued until the capacity retention fell below 80%. Notably, all of the cells demonstrated acceptable durability, maintaining over 80% capacity after at least 170 cycles. The comparative analysis indicates that the cell durability ranks as follows: Sn-LATP (510 cycles) > V-LATP (450 cycles) > Co-LATP (275 cycles) > Zr-LATP (240 cycles) > Cu-LATP (200 cycles) > 0-LATP (170 cycles). Compared to other dopants, the ionic radius of Sn^4+^ (0.69 Å) is closer to that of Ti^4+^ (0.605 Å). Therefore, the crystal structure does not experience excessive lattice distortion [59]. Furthermore, Sn^4+^ with higher Pauling electronegativity (1.96) can improve the stability of the NASICON framework [42,59]. Consequently, the cell equipped with the Sn-LATP CSE demonstrated optimal performance and maintained high Coulombic efficiency (>99.0%) over 510 cycles. These data also reveal the potential of these CSEs for improved electrochemical stability against Li metal electrodes (since Li metal foil was used as the counter electrodes for the LTO-supported CSE cells). After cell testing, no cation reduction reaction is observed. This phenomenon is likely attributed to the presence of the polymer phase in the CSE. The polymer phase plays a crucial role in the system to encapsulate the LATP particles, thus forming a protective barrier that isolates the LATP particles from direct contact with the lithium metal. The ceramic fillers enhance the mechanical strength of the CSEs, acting as the capping layers to suppress Li dendrite formation. Sn doping in LATP has been confirmed as an agreeable strategy to improve the CSE performance, which is highly compatible with both Li metal and LTO electrodes, enabling superior rate capability and desirable cycling stability of the solid-state cell.

## 4. Conclusions

This study demonstrates the proper selection of metal-doped NASICON-type LATP SSE in enabling LTO anodes with high rate capability and great cycling performance. The Li^+^-conducting LATP particles were successfully synthesized through a modified sol–gel method followed by thermal calcination. Five types of dopant (i.e., Co, Cu, Sn, V, and Zr) clearly influenced the substitution of Al for Ti, with the dopant type serving as a crucial factor in tuning the Li^+^ conductivity and the ESR of the LTO-supported CSE cells. The CSE containing the Sn-LATP particles exhibited an ionic conductivity of 1.88 × 10^−4^ S cm^−1^ at ambient temperature. This enhanced conductivity could be attributed to the alteration in lattice parameters and the Li^+^ transport pathways due to the Sn doping. The solid-state cell equipped with the LTO-supported CSE containing Sn-LATP fillers demonstrated both high rate capability at 5 C (with a capacity retention of 86% compared to the value measured at 0.2 C) and superior cycling stability, maintaining 80% of the initial capacity after 510 cycles. The close ionic radii of Sn^4+^ and Ti^4+^ prevent excessive lattice distortion, and the higher Pauling electronegativity of Sn^4+^ enhances the stability of the NASICON framework, leading to the optimal cell performance. These findings indicate that the CSE, composed of Sn-LATP ceramics, PVDF-HFP, and LiTFSI, is highly promising for use in SSLBs featuring high performance and high durability.

## Data Availability

Data are contained within the article and supplementary materials.

## Supplementary Materials

The following supporting information can be downloaded at: https://www.mdpi.com/article/10.3390/polym16091251/s1, Figure S1: EIS data of Li||CSE (with 0-LATP)||LTO cell.
