# Supplementary material for: Metal-Doped NASICON/Polymer Composite Solid Electrolyte for Lithium Titania Anode in Lithium-Ion Batteries
Source: Polymers (Basel). 2024 Apr 30;16(9):1251. doi: 10.3390/polym16091251 (PMC11085400; doi:10.3390/polym16091251)
Supplement: Supplementary file 1 [file polymers-16-01251-s001.zip › polymers-2927795-supplementary.pdf]

Supplementary Information

Metal-Doped NASICON/Polymer Composite Solid Electrolyte for Lithium Titania Anode of Li-ion Battery

Chien-Te Hsieh, Tzu-Shaing Cho, Jeng-Kuei Chang, Jagabandhu Patra

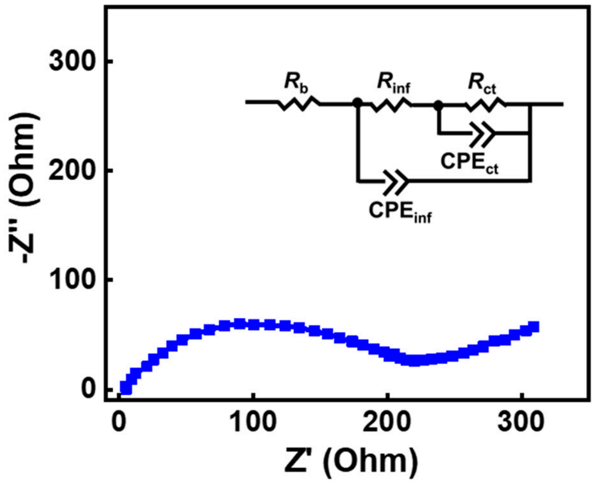

Figure S1. EIS data of Li||CSE (with 0-LATP)||LTO cell.

Note: The equivalent circuit used to fit the EIS data are shown in the figure inset. The R_b, R_{inf}, and R_{ct} are bulk electrolyte resistance, electrolyte/electrode interfacial resistance, and charge-transfer resistance, respectively. The CPE_{inf} and CPE_{ct} are interfacial and charge-transfer constant-phase elements, respectively.
